# Complete genome sequence of *Rhodothermaceae* bacterium RA with cellulolytic and xylanolytic activities

**DOI:** 10.1007/s13205-018-1391-z

**Published:** 2018-08-13

**Authors:** Kok Jun Liew, Seng Chong Teo, Mohd Shahir Shamsir, Rajesh Kumar Sani, Chun Shiong Chong, Kok-Gan Chan, Kian Mau Goh

**Affiliations:** 1 Faculty of Science, Universiti Teknologi Malaysia, 81300 Skudai, Johor Malaysia; 2 Department of Chemical and Biological Engineering, South Dakota School of Mines and Technology, Rapid City, USA; 3 Institute of Biological Sciences, Faculty of Science, University of Malaya, 50603 Kuala Lumpur, Malaysia; 4 International Genome Centre, Jiangsu University, Zhenjiang, 212013 People’s Republic of China

**Keywords:** Cellulase, Xylanase, Halophile, *Rhodothermaceae*, *Rhodothermus*

## Abstract

*Rhodothermaceae* bacterium RA is a halo-thermophile isolated from a saline hot spring. Previously, the genome of this bacterium was sequenced using a HiSeq 2500 platform culminating in 91 contigs. In this report, we report on the resequencing of its complete genome using a PacBio RSII platform. The genome has a GC content of 68.3%, is 4,653,222 bp in size, and encodes 3711 genes. We are interested in understanding the carbohydrate metabolic pathway, in particular the lignocellulosic biomass degradation pathway. Strain RA harbors 57 glycosyl hydrolase (GH) genes that are affiliated with 30 families. The bacterium consists of cellulose-acting (GH 3, 5, 9, and 44) and hemicellulose-acting enzymes (GH 3, 10, and 43). A crude cell-free extract of the bacterium exhibited endoglucanase, xylanase, β-glucosidase, and β-xylosidase activities. The complete genome information coupled with biochemical assays confirms that strain RA is able to degrade cellulose and xylan. Therefore, strain RA is another excellent member of family *Rhodothermaceae* as a repository of novel and thermostable cellulolytic and hemicellulolytic enzymes.

**Electronic supplementary material:**

The online version of this article (10.1007/s13205-018-1391-z) contains supplementary material, which is available to authorized users.

## Introduction

Lignocellulosic biomass is mainly composed of cellulose, hemicellulose, and lignin. The degradation of these complex structures involves a series of enzymes that work synergistically. Enzymes responsible for cellulose degradation are cellulase, including endoglucanase (EC 3.2.1.4), β-glucosidase (EC 3.2.1.21), and exoglucanase (EC 3.2.1.91). These enzymes are classified into glycosyl hydrolase (GH) families GH1, GH3, GH5, GH6, GH7, GH8, GH9, GH12, GH45, and GH48 (Bohra et al. 2018). Hemicellulose is enzymatically hydrolyzed by a mixture of enzymes, including xylanase (EC 3.2.1.8), β-galactosidase (EC 3.2.1.23), β-mannosidase (EC 3.2.1.25), β-glucuronidase (EC 3.2.1.31), β-xylosidase (EC 3.2.1.37), β-d-fucosidase (EC 3.2.1.38), and α-l-arabinofuranosidase (EC 3.2.1.55). These hemicellulases are mainly found in the GH families GH2, GH10, GH11, GH16, GH26, GH30, GH31, GH36, GH43, GH51, GH74, and GH95 (Bohra et al. 2018). Enzymes such as laccase, lignin peroxidase, and manganese peroxidase are also crucial in lignocellulosic biomass degradation, in particular, the lignin moiety. The majority of these enzymes can be found in the Auxiliary Activities (AA) families listed in the CAZy database (Lombard et al. 2013).

Family *Rhodothermaceae* has not been studied extensively for lignocellulose degradation. Members of this family are rod- or cocci-shaped, stain gram negative, non-sporulating, chemoorganotrophic aerobes, and are known to produce pigments (Park et al. 2014). Currently members of family *Rhodothermaceae* consists of six genera: *Rhodothermus* (Alfredsson et al. 1988; Marteinsson et al. 2010), *Salinibacter* (Antón et al. 2002; Makhdoumi-Kakhki et al. 2012), *Salisaeta* (Vaisman and Oren 2009), *Longimonas* (Xia et al. 2015), *Longibacter* (Xia et al. 2016), and *Natronotalea* (Sorokin et al. 2017). Members of the genera *Rubricoccus* and *Rubrivirga* were previously affiliated to family *Rhodothermaceae* (Park et al. 2011, 2013; Goh et al. 2016), but have recently been reassigned and classified as members of a new family, family *Rubricoccaceae* (Munoz et al. 2016). Genome sequences are available from representatives of hall genera of family *Rhodothermaceae* except for any representative of genus *Natronotalea*. To date, complete genome sequences of two representatives of this family, namely, *Rhodothermus marinus* and *Salinibacter ruber*, are available. Strain RA is a halo-thermophile (optimum growth at 2% w/v NaCl, 50 °C) which was isolated from a saline hot spring located on Langkawi Island, Malaysia (6°25′22″N, 99°48′49″E) (Goh et al. 2016; Chan et al. 2017). Due to the low DNA–DNA similarity as measured by Genome-to-Genome Distance Calculator (GGDC), 16S rRNA gene similarity, and the housekeeping genes to the other members of the family *Rhodothermaceae*, strain RA has been assigned as an unclassified taxon of the family *Rhodothermaecae*, order *Bacteroidetes Order II. Incertae sedis*, and phylum *Bacteroidetes*. Detailed the low 16S rRNA gene sequence similarity of strain RA (89%) compared to *Rhodothermus spp., Salisaeta longa, Longibacter salinarum, Longimonas halophila, Salinibacter spp*., and *Natronotalea spp*. suggests that it should be included as a new species in a newly created genus status. The genome of strain RA was initially sequenced in 2015 using a HiSeq 2500 platform and assembled into 91 contigs (Goh et al. 2016). Here, we report on the full genome using a PacBio single-molecule sequencing platform and in the presence of lignocellulose biomass-degrading enzymes.

## Materials and methods

### Genome sequencing, assembly, and annotation

Strain RA (KCTC 62031) was originally isolated from a hot spring located in Langkawi, a Malaysian island (Goh et al. 2016). The cells were resuscitated from 20% (*v*/*v*) glycerol stock, grown on marine agar plates (pH 7.5), and incubated at 50 °C for 48 h. Colonies on the agar plates were scraped and DNA extraction using a Quick-DNA™ Miniprep Plus kit (Zymo Research, Irvine, USA). The extracted genomic DNA was analyzed using a NanoDrop 1000 spectrophotometer and Qubit® 3.0 fluorometer (Thermo Scientific, Waltham, USA) to check its purity (*A*_260/280_ ratio) and concentration. The genomic DNA was then constructed into a 20-kb SMRTbell™ template library and sequenced using a PacBio RSII sequencing platform (Pacific Biosciences, CA, USA). The resulting sequence was assembled using a PacBio Hierarchical Genome Assembly Process (HGAP) algorithm version 2 (Chin et al. 2013). The final assembled genome was analyzed and annotated using the NCBI Prokaryotic Genome Annotation Pipeline (PGAP) version 2.10 (Tatusova et al. 2016). (Tatusova et al. 2016). A cluster of orthologous genes (COG) (Tatusov et al. 2003) was carried out for gene function analysis. Kyoto Encyclopedia of Genes and Genomes (KEGG) (Kanehisa and Goto 2000; Kanehisa et al. 2017) was utilized for pathway analysis. The GH proteins from strain RA were further classified using dbCAN HMMs 5.0 (Yin et al. 2012), and the results were validated with the annotations available online in the Carbohydrate-Active Enzymes (CAZy) database (Lombard et al. 2013).

### Enzymatic assay of bacterial whole cell lysate

To induce production of both cellulolytic and xylanolytic enzymes, strain RA was grown in marine broth supplemented with both 0.1% (*w*/*v*) carboxylmethyl cellulose (CMC) and 0.1% (*w*/*v*) beechwood xylan. After 72-h incubation, crude enzymes were extracted from the cells and dialyzed against 20-mM sodium phosphate buffer (pH 8) using a 10K MWCO SnakeSkin™ dialysis tubing (Thermo Scientific, Waltham, USA). Unless specified, all enzyme assays were carried out at 50 °C, pH 8 for 15 min by incubating 0.1 mL of crude enzymes with 1 mL of substrate, and subsequently measured using a 7300 Vis spectrophotometer (Jenway, Staffordshire, UK) with the wavelength adjusted to 540 nm (for reducing sugar detection by DNS assay), or at 405 nm (for detection of p-nitrophenol released from the artificial substrates). Substrates tested included Avicel^®^, CMC, beechwood xylan, p-nitrophenyl-β-d-glucopyranoside (pNPG), p-nitrophenyl-β-d-xylopyranoside (pNPX), cellodextrins (cellobiose to celloheptaose, C2–C7), and xylodextrins (xylobiose to xylohexaose, X2–X6). The post-reaction products were determined using an Agilent 1260 Infinity High-Performance Liquid Chromatography, coupled with an Agilent 385-Evaporative Light Scattering Detector (Agilent Technologies, Santa Clara, USA) and a Rezex RSO-Oligosaccharide Ag + column (Phenomenex Inc, Torrance, USA).

## Results and discussion

### Genome features of strain RA

**Fig. 1:**
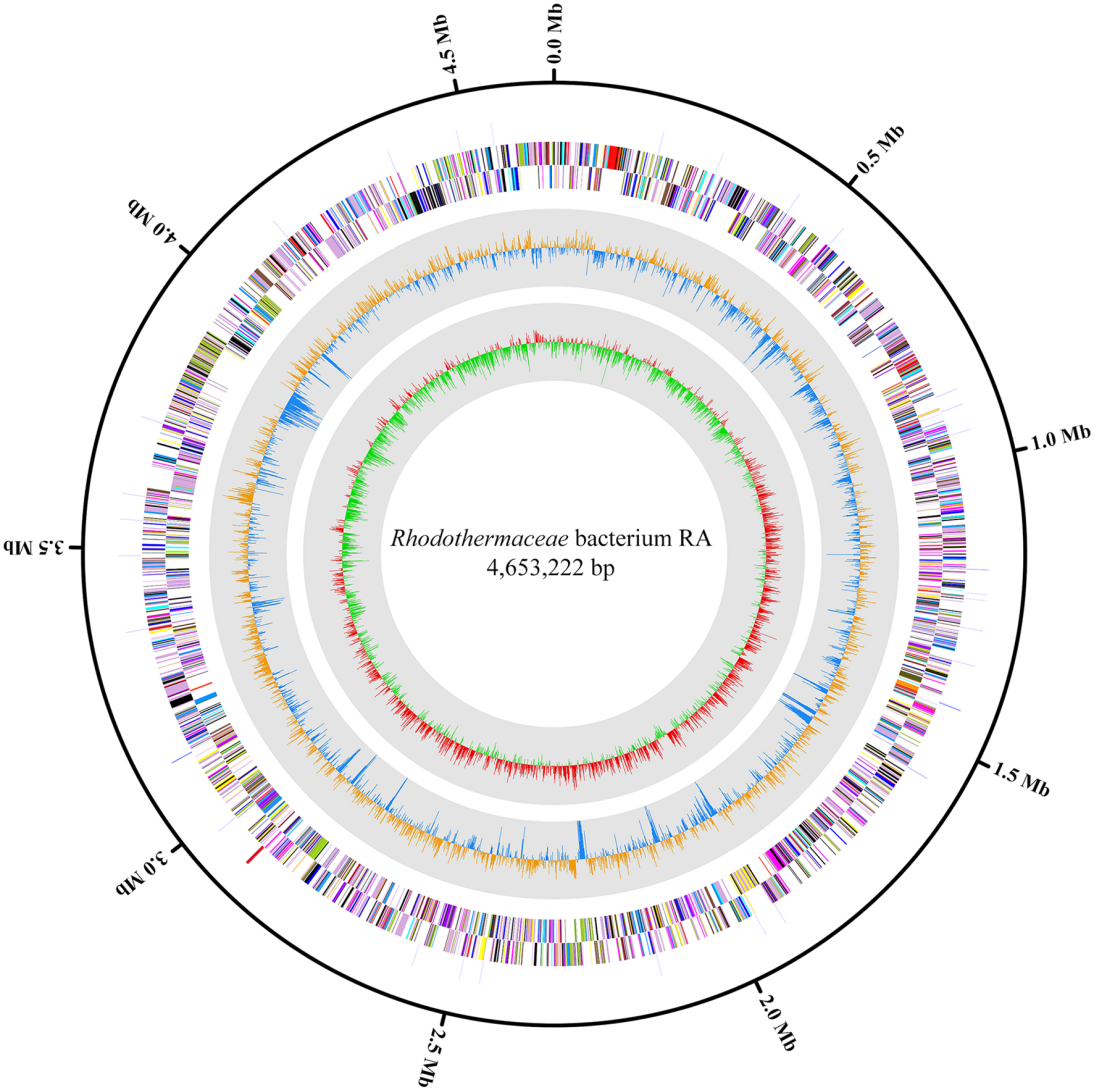
Circular genome map of *Rhodothermaceae* bacterium RA. From the outermost circle to the center: RNA genes (rRNA, tRNA, and ncRNA), Reverse CDS, Forward CDS, GC skew, and GC ratio

As strain RA is most likely a new genus of the family *Rhodothermaceae*, we resequenced the genome to fill in the gaps, as well as to confirm the orientation or order of contigs present in the draft genome. The PacBio RSII sequencer was able to close the gaps found in the earlier draft genome. An additional 71 CDS were also identified in the newly assembled genome. The complete genome of this bacterium has been deposited in GenBank under accession number CP020382.1. The circular chromosome of 4,653,222 bp (132x coverage) had a GC content of 68.3%, and based on NCBI PGAP (Fig. 1), the genome encoded 3,711 genes, which included 3,506 protein-coding sequences (CDS), 155 pseudogenes, 3 rRNAs, 44 tRNAs, and 3 ncRNAs. Moreover, a total of 1730 genes (46.6% of the total genes) from strain RA are annotated as hypothetical protein or uncharacterized protein due to their low sequence similarities to the existing database. A total of 3417 genes are annotated into different functional categories according to COG analysis (Table 1). Based on KEGG (Entry number T04780), strain RA possessed all genes for most of the carbohydrate metabolism pathways.

**Table 1:** COG functional categories of *Rhodothermaceae* bacterium RA

COG functional categories	Count	Proportion (%)
Information storage and processing		
*J*—translation, ribosomal structure, and biogenesis	147	4.30
*A*—RNA processing and modification	1	0.03
*K*—transcription	124	3.63
*L*—replication, recombination, and repair	142	4.16
*B*—chromatin structure and dynamics	3	0.09
Cellular processes and signaling		
*D*—cell cycle control, cell division, and chromosome partitioning	25	0.73
*Y*—nuclear structure	0	0.00
*V*—defence mechanisms	43	1.26
*T*—signal transduction mechanisms	180	5.27
*M*—cell wall/membrane/envelope biogenesis	209	6.12
*N*—cell motility	38	1.11
*Z*—cytoskeleton	1	0.03
*W*—extracellular structures	1	0.03
*U*—intracellular trafficking, secretion, and vesicular transport	44	1.29
*O*—posttranslational modification, protein turnover, and chaperones	122	3.57
Metabolism		
*C*—energy production and conversion	148	4.33
*G*—carbohydrate transport and metabolism	183	5.36
*E*—amino acid transport and metabolism	237	6.94
*F*—nucleotide transport and metabolism	70	2.05
*H*—coenzyme transport and metabolism	92	2.69
*I*—lipid transport and metabolism	77	2.25
*P*—inorganic ion transport and metabolism	195	5.71
*Q*—secondary metabolites biosynthesis, transport, and catabolism	58	1.70
Poorly characterized		
*R*—general function prediction only	0	0.00
*S*—function unknown	1277	37.37

**Fig. 2:**
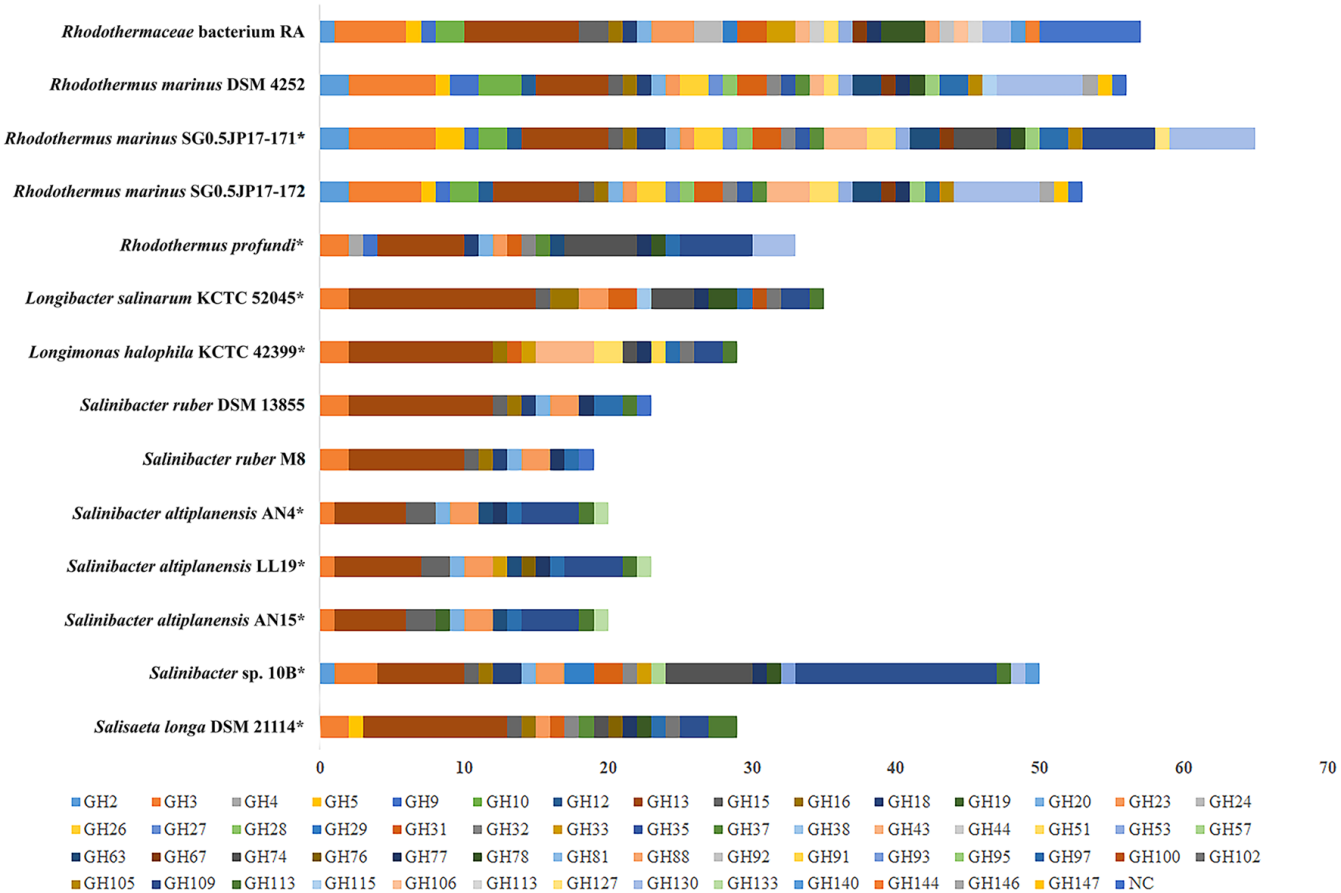
Distribution and predicted numbers of GH in the genome *Rhodothermaceae* bacterium RA and other bacteria strains of the same genus. *indicates draft genome sequences

Figure 2 illustrates the distribution of glycosyl hydrolases (GHs) in the genome of strain RA and other genera affiliated with family *Rhodothermaceae* which includes *Rhodothermus marinus* DSM 4252 (CP001807.1), *R. marinus* SG0.5JP17-171 (GCA_000565305.1), and *R. marinus* SG0.5JP17-172 (CP003029.1) that exhibit average 54 GH sequences placed in 31 GH families. Strain RA has a distribution of GH sequences similar to *R. marinus*, with 57 GHs that are affiliated to 30 GH families. The total number of GHs annotated in the genome of strain RA is higher than other genera of the family *Rhodothermaceae*. For instance, *Rhodothermus profundi* (GCA_900142415.1), *Longibacter salinarum* (GCA_002554795.1), *Longimonas halophila* (GCA_002554705.1), and *Salisaeta longa* (GCA_000419585.1) have 29–35 GHs (Fig. 2). Most *Salinibacter* spp. (CP000159.1/FP565814.1/GCA_002894605.1/ GCA_002894625.1/GCA_002894645.1) have around 21 sequences grouped into 17 different GH families, except for *Salinibacter* sp. 10B (GCA_002954405.1), which has 50 GHs across 22 families.

Several genes present in strain RA are annotated as GH enzymes related to cellulose and hemicellulose degradation (Table 2). These sequences include a GH2 β-galactosidase (NCBI locus tag: AWN76_014570), GH3 β-glucosidase (AWN76_006445), GH5 endoglucanase (AWN76_009395), GH9 endoglucanase (AWN76_010685), GH10 xylanase (AWN76_003690 and AWN76_008205), GH43 β-xylosidase (AWN76_012335), GH53 endo-β-1,4-galactanase (AWN76_017855), and GH92 α-mannosidase (AWN76_002955). Interestingly, these enzymes have low identities to other counterpart sequences available in the NCBI database (57–73% identity). In addition, these sequences exhibited low similarity to *Rhodothermus* spp. counterparts, a clear indication of the novelty of enzymes from strain RA. A putative sequence (AWN76_009940) was annotated as glycoside hydrolase. The AWN76_009940 protein sequence consists of a typical GH16 domain as determined using InterProScan, and it is 75% identical to laminarinase (endo-1,3(4)-β-glucanase; PDB id: 3ILN_A) which originates from *R. marinus* (Bleicher et al. 2011). Another sequence (AWN76_008195) is annotated as a hypothetical protein but putatively functions as an endoglucanase associated with GH44. The protein sequence of AWN76_008195 is 56% identical to endoglucanase J of *Ruminiclostridium thermocellum* (Ahsan et al. 1996). Other than GHs, some of the genes from strain RA are also assigned to other CAZy families, including 60 glycosyl transferases (GTs), 4 polysaccharide lyases (PLs), 8 carbohydrate esterases (CEs), 16 carbohydrate-binding modules (CBMs), and 7 auxiliary activities (AA) affiliated enzymes. According to CAZy, AA consists of two groups of enzymes (ligninolytic enzymes and lytic polysaccharide mono-oxygenases), that are responsible for lignin breakdown, as well as the hydrolysis of polysaccharide. Therefore, it is likely that these enzymes in strain RA may work cooperatively with GHs to efficiently degrade the lignocellulosic biomass.

**Table 2:** List of potential lignocellulolytic enzymes from *Rhodothermaceae* bacterium RA

CAZyme families	Annotation	locus_tag	RefSeq accession number	Closest Sequence	Identity (%)
GH2	β-glucosidase	AWN76_014570	ARA94254.1	glycoside hydrolase of *Gemmatimonadetes* bacterium	65
GH3	β-glucosidase	AWN76_006445	ARA95045.1	β-glucosidase BglX of *Rhodothermus marinus*	69
GH5	Endoglucanase	AWN76_009395	ARA93352.1	glycoside hydrolase of *Pedobacter* sp. *V48*	57
GH9	Endoglucanase	AWN76_010685	ARA95103.1	glycoside hydrolase family 9 of *Gemmatimonas* sp	73
GH10	Xylanase	AWN76_003690	ARA92359.1	glycoside hydrolase of *Rhodothermus marinus*	73
GH10	Xylanase	AWN76_008205	ARA95075.1	endo-1,4-beta-xylanase of *Candidatus Solibacter usitatus*	62
GH43	β-xylosidase	AWN76_012335	ARA93868.1	glycoside hydrolase of *Parapedobacter composti*	60
GH53	endo-β-1,4-galactanase	AWN76_017855	ARA94834.1	arabinogalactan endo-1,4-β-galactosidase of *Rhodothermus marinus*	60
GH92	α-mannosidase	AWN76_002955	ARA92237.1	α-mannosidase of *Spirosoma* sp. 209	58
GH16	Glycosyl hydrolase	AWN76_009940	ARA93444.1	laminarinase of *Rhodothermus marinus*	75
GH44	Hypothetical protein	AWN76_008195	ARA93141.1	endoglucanase J of *Ruminiclostridium thermocellum*	56
AA2	Catalase/peroxidase HPI	AWN76_014060	ARA94166.1	Catalase/peroxidase HPI of *alpha Proteobacterium*	74
AA3	GMC family oxidoreductase	AWN76_001955	ARA92052.1	GMC family oxidoreductase of *Rhodothermus marinus*	70
AA3	GMC family oxidoreductase	AWN76_003120	ARA92263.1	GMC family oxidoreductase of *Rhodothermus marinus*	70
AA3	Patatin	AWN76_007050	ARA92944.1	Patatin-like phospholipase family protein of *Catalinimonas alkaloidigena*	61
AA3	GMC family oxidoreductase	AWN76_011750	ARA93772.1	GMC family oxidoreductase of *Rhodothermus marinus*	67
AA12	Sorbosone dehydrogenase	AWN76_005825	ARA92731.1	Sorbosone dehydrogenase of *Rhodothermus marinus*	64
AA12	Sorbosone dehydrogenase	AWN76_011490	ARA95111.1	Sorbosone dehydrogenase of *Phormidesmis priestleyi*	64

### Cellulolytic and xylanolytic potential of strain RA

Table 3 summarizes the results of both colorimetric assays and HPLC analysis. In brief, the cell-free crude enzymes of strain RA are active on the following substrates: CMC, beechwood xylan, pNPG, pNPX, cellodextrins (C2–C7), and xylodextrins (X2–X6). Under the current experimental setup, the crude enzymes of strain RA exhibit 0.41-U/mL endoglucanase, 0.02-U/mL β-glucosidase, 1.43-U/mL xylanase, and 0.17-U/mL β-xylosidase activities. The crude enzymes were not active against Avicel^®^ suggesting the absence of exoglucanase activity, confirming the absence of such an enzyme from genome annotation. Similarly, exoglucanase gene is also absent from members of genus *Rhodothermus*, the closest relative of strain RA. Many of the distant thermophilic bacteria (*Thermotoga maritima, Dictyoglomus turgidum*, and *Thermomonospora curvata*) (Chertkov et al. 2011; Singh et al. 2015; Brumm et al. 2016), also lack of exoglucanase gene. Other thermophiles (*Ruminiclostridium thermocellum, Caldicellulosiruptor* spp., and *Thermobifida fusca*) produce exoglucanase (Caspi et al. 2008; Blumer-Schuette et al. 2010; Sheng et al. 2016).

**Table 3:** Hydrolysis of various substrates by *Rhodothermaceae* bacterium RA crude enzymes

Substrate	Spectrophotometric analysis^a,b^ (Unit/mL)	HPLC analysis						
		Substrate depletion^c^ (%)	Product formation (µg/mL)					
			Glucose	C2	C3	C4	Xylose	X2
Avicel	0.000 ± 0.000^a^	–	0	0	0	0	–	–
CMC	0.411 ± 0.011^a^	–	82	39	18	0	–	–
Xylan	1.428 ± 0.007^a^	–	–	–	–	–	1397	41
PNPG	0.019 ± 0.001^b^	–	–	–	–	–	–	–
PNPX	0.173 ± 0.001^b^	–	–	–	–	–	–	–
C2	–	16.86	318	–	–	–	–	–
C3	–	30.06	314	810	–	–	–	–
C4	–	99.28	405	4928	464	–	–	–
C5	–	100.00	430	3042	3909	0	–	–
C6	–	100.00	359	3354	2185	229	–	–
C7	–	100.00	306	1230	494	58	–	–
X2	–	95.06	–	–	–	–	4233	–
X3	–	100.00	–	–	–	–	3958	419
X4	–	100.00	–	–	–	–	3786	368
X5	–	100.00	–	–	–	–	3392	240
X6	–	100.00	–	–	–	–	3276	193

– indicates not available. C2–C7 indicate cellobiose to celloheptaose, respectively. X2–X6 indicate xylobiose to xylohexaose, respectively

^a^Reading taken at wavelength 540 nm (DNS assay). One unit (U) of enzyme activity was defined as the enzyme amount that can liberate 1 µmol of reducing sugar per min per mL under assay condition

^b^Reading taken at wavelength 405 nm. One unit (U) of enzyme activity was defined as the enzyme amount that can liberate 1 µmol of p-nitrophenol per min per mL under assay condition

^c^Calculated using formula: $\frac{{{\text{Initial}}\,{\text{amount}}\,{\text{of}}\,{\text{substrate}} - {\text{amount}}\,{\text{of}}\,{\text{substrate}}}}{{{\text{Initial}}\,{\text{amount}}\,{\text{of}}\,{\text{substrate}}}} \times {\text{1}}00\%$

Crude enzymes were assayed with CMC and xylan and the end-products were analyzed using HPLC. The crude enzyme was found to hydrolyze CMC and C2–C7 to glucose, cellobiose, cellotriose, and cellotetraose, whereas the major degradation products from xylan and X2–X6 were xylose and xylobiose. In general, more total sugars were released from xylan than CMC (Table 3). Besides, cell-free crude enzymes efficiently hydrolyzed longer chains substrates (C5–C7, X3–X6), but the activities gradually dropped for shorter chains substrates (C2–C4, X2). It is hypothesized that under current experiment condition, supplementation of xylan and CMC to the growth culture favoured the expression of endoglucanase and xylanase activities, which are specific to longer chains substrates. Although the activities were detected for β-glucosidase and β-xylosidase, both enzymes were likely not over-expressed by the induction of xylan and CMC.

Comparison of strain RA enzyme activities with a list of other lignocellulolytic bacteria is summarized in Appendix A. *Cellvibrio mixtus, Jonesia denitrificans*, and *Gracilibacillus* sp. TSCPVG are outstanding xylanase producers (Giridhar and Chandra 2010; Nawel et al. 2011; Wu and He 2015). *Clostridium thermocellum* is an excellent bacterium for endoglucanase production (Mori 1992). At the current time, data on lignocellulose saccharification studies using members of the family *Rhodothermaceae* are limited. In addition, currently, the ability to degrade cellulose and hemicellulose is known to be restricted to members of the genus *Rhodothermus*. According to a study by Dahlberg et al. (1993), *R. marinus* DSM 4252 exhibited < 0.03-U/mL endoglucanase, 1.98-U/mL β-glucosidase, 1.14-U/mL xylanase, and 4.08-U/mL β-xylosidase activities after 24-h growth in modified M162 medium supplemented with 0.5% (*w*/*v*) xylan in a 2.5-L bioreactor (note: unit conversion from nkat/mL to U/mL). Moreover, other reports related to *Rhodothermus* spp. enzymes such as endoglucanase, xylanase, endo-1,4-β-mannosidase, and α-l-arabinofuranosidase have also been reported (Karlsson et al. 1997; Halldórsdóttir et al. 1998; Politz et al. 2000; Gomes et al. 2000). All these enzymes have been purified, characterized, and were reported to be active and thermostable. Altogether, *Rhodothermus* is known as an interesting genus for lignocellulose degradation. In conclusion, the current work reports the improved genome sequence of strain RA. In addition to members of genus *Rhodothermus*, strain RA is yet another excellent candidate in the family *Rhodothermaceae* which possess a repertoire of novel and thermostable cellulolytic and hemicellulolytic enzymes.

## Electronic supplementary material

Below is the link to the electronic supplementary material.

Supplementary material 1 (DOCX 22 KB)
