# Supplementary material for: Complete genome sequence of Rhodothermaceae bacterium RA with cellulolytic and xylanolytic activities
Source: 3 Biotech. 2018 Aug 13;8(8):376. doi: 10.1007/s13205-018-1391-z (PMC6087703; doi:10.1007/s13205-018-1391-z)
Supplement: Supplementary file 1 — Supplementary material 1 (DOCX 22 KB) [file 13205_2018_1391_MOESM1_ESM.docx]

**Appendix A** Comparison of exoglucanase, endoglucanase, β-glucosidase, xylanase, and β-xylosidase activities of different bacterial strains.

|  | Enzyme Activity (U/mL) | | | | |  |
| --- | --- | --- | --- | --- | --- | --- |
|  | Exoglucanase | Endoglucanase | β-glucosidase | Xylanase | β-xylosidase | References |
| *Rhodothermaceae* bacterium RA | - | 0.41 | 0.02 | 1.43 | 0.17 | This study |
| *Rhodothermus marinus* DSM 4252 | <0.06 | <0.03 | 1.98 | 1.14 | 4.08 | (Dahlberg et al. 1993) |
| *Acinetobacter anitratus* | - | 0.48 | 0.24 | - | - | (Ekperigin 2007) |
| *Arthrobacter* sp. Am13 | - | 0.06 | - | - | - | (Liang et al. 2014) |
| *Bacillus megaterium* | 0.09 | 0.10 | - | - | - | (Beukes and Pletschke 2006) |
| *Bacillus pumilus* BpCRI 6 | - | 1.90 | 1.20 | - | - | (Kotchoni et al. 2003) |
| *Bacillus pumilus* EB3 | 0.01 | 0.08 | 0.04 | - | - | (Ariffin et al. 2006) |
| *Bacillus pumilus* EWBCM1 | - | 0.59 | - | - | - | (Shankar and Isaiarasu 2011) |
| *Bacillus pumilus* Kd101 TUC-EEAOC | - | 0.32 | - | - | - | (Dantur et al. 2015) |
| *Bacillus* sp. |  | 0.26 | - | - | - | (Li et al. 2008) |
| *Bacillus* sp. BMP01 | - | 0.25 | - | 0.21 | - | (Tsegaye et al. 2018) |
| *Bacillus* sp. DUSELR12 | - | 0.12 | - | - | - | (Rastogi et al. 2009) |
| *Bacillus* sp. SMIA-2 | 0.83 | 0.29 | - | - | - | (Ladeira et al. 2015) |
| *Bacillus subtilis* AS3 | - | 0.43 | - | - | - | (Deka et al. 2011) |
| *Bacillus subtilis* CK-2 | - | 0.26 | - | - | - | (Aa et al. 1994) |
| *Branhamella* sp. | - | 2.56 | 0.34 | - | - | (Ekperigin 2007) |
| *Brevibacillus* sp. DUSELG12 | 0.03 | 0.02 | - | - | - | (Rastogi et al. 2009) |
| *Brevibacillus* sp. JXL | 0.02 | - | - | - | - | (Liang et al. 2009) |
| *Cellvibrio mixtus* J3-8 | - | - | - | 10.10 | - | (Wu and He 2015) |
| *Clostridium thermocellum* YM4 | - | 6.70 | - | - | - | (Mori 1992) |
| *Geobacillus* sp. | 0.04 | 0.05 | - | - | - | (Rastogi et al. 2009) |
| *Geobacillus thermoleovorans* T4 | - | 0.01 | - | - | - | (Tai et al. 2004) |
| *Gracilibacillus* sp. TSCPVG | - | - | - | 18.44 | 1.01 | (Giridhar and Chandra 2010) |
| *Jonesia denitrificans* | - | - | - | 10.81 | - | (Nawel et al. 2011) |
| *Klebsiella oxytoca* Kd70 TUC-EEAOC | - | 0.22 | - | - | - | (Dantur et al. 2015) |
| *Paenibacillus terrae* ME27-1 | - | 2.08 | - | - | - | (Liang et al. 2014) |
| *Pseudomonas* sp. HP207 | - | 1.43 | - | - | - | (Sheng et al. 2012) |
| *Ruminiclostridium thermocellum* M3 | 0.17 | 0.19 | 0.33 | - | - | (Sheng et al. 2016) |
| *Streptomyces drozdowiczii* | - | 0.59 | - | - | - | (de Lima et al. 2005) |
| *Streptomyces* sp. (Strain J2) | - | 0.43 | - | - | - | (Jaradat et al. 2008) |
| *Streptomyces* sp. SLBA-08 | - | 1.11 | - | - | - | (Macedo et al. 2013) |
| *Streptomyces viridobrunneus* SCPE-09 | - | 2.00 | - | - | - | (Da Vinha et al. 2011) |

Note: The production of cellulolytic enzymes was not optimized for some of the bacteria, including this study. The references for this appendix are listed below.

**References for this appendix**

Aa K, Flengsrud R, Lindahl V, Tronsmo A (1994) Characterization of production and enzyme properties of an endo-β-1, 4-glucanase from *Bacillus subtilis* CK-2 isolated from compost soil. Antonie Van Leeuwenhoek 66:319–326

Ariffin H, Abdullah N, Umi Kalsom M, Shirai Y, Hassan MA (2006) Production and characterization of cellulase by *Bacillus pumilus* EB3. Int J Eng Technol 3:47–53

Beukes N, Pletschke BI (2006) Effect of sulfur-containing compounds on *Bacillus* cellulosome-associated ‘CMCase’and ‘Avicelase’activities. FEMS Microbiol Lett 264:226–231

Da Vinha FNM, Gravina-Oliveira MP, Franco MN, Macrae A, da Silva Bon EP, Nascimento RP, Coelho RRR (2011) Cellulase production by *Streptomyces viridobrunneus* SCPE-09 using lignocellulosic biomass as inducer substrate. Appl Biochem Biotechnol 164:256–267

Dahlberg L, Holst O, Kristjansson JK (1993) Thermostable xylanolytic enzymes from *Rhodothermus marinus* grown on xylan. Appl Microbiol Biotechnol 40:63–68

Dantur KI, Enrique R, Welin B, Castagnaro AP (2015) Isolation of cellulolytic bacteria from the intestine of *Diatraea saccharalis* larvae and evaluation of their capacity to degrade sugarcane biomass. AMB Express 5:15

de Lima ALG, do Nascimento RP, da Silva Bon EP, Coelho RRR (2005) *Streptomyces drozdowiczii* cellulase production using agro-industrial by-products and its potential use in the detergent and textile industries. Enzyme Microb Technol 37:272–277

Deka D, Bhargavi P, Sharma A, Goyal D, Jawed M, Goyal A (2011) Enhancement of cellulase activity from a new strain of *Bacillus subtilis* by medium optimization and analysis with various cellulosic substrates. Enzyme Res 2011:

Ekperigin M (2007) Preliminary studies of cellulase production by *Acinetobacter anitratus* and *Branhamella* sp. Afr J Biotechnol 6:28–33

Giridhar PV, Chandra T (2010) Production of novel halo-alkali-thermo-stable xylanase by a newly isolated moderately halophilic and alkali-tolerant *Gracilibacillus* sp. TSCPVG. Process Biochem 45:1730–1737

Jaradat Z, Dawagreh A, Ababneh Q, Saadoun I (2008) Influence of culture conditions on cellulase production by *Streptomyces* sp.(strain J2). Jordan J Biol Sci 1:141–146

Kotchoni Od, Shonukan O, Gachomo W (2003) *Bacillus pumilus* BpCRI 6, a promising candidate for cellulase production under conditions of catabolite repression. Afr J Biotechnol 2:140–146

Ladeira SA, Cruz E, Delatorre AB, Barbosa JB, Leal Martins ML (2015) Cellulase production by thermophilic *Bacillus* sp: SMIA-2 and its detergent compatibility. Electron J Biotechnol 18:110–115

Li W, Zhang W-W, Yang M-M, Chen Y-L (2008) Cloning of the thermostable cellulase gene from newly isolated *Bacillus subtilis* and its expression in *Escherichia coli*. Mol Biotechnol 40:195–201

Liang Y-L, Zhang Z, Wu M, Wu Y, Feng J-X (2014) Isolation, screening, and identification of cellulolytic bacteria from natural reserves in the subtropical region of China and optimization of cellulase production by *Paenibacillus terrae* ME27-1. BioMed Res Int 2014:

Liang Y, Yesuf J, Schmitt S, Bender K, Bozzola J (2009) Study of cellulases from a newly isolated thermophilic and cellulolytic *Brevibacillus* sp. strain JXL. J Ind Microbiol Biotechnol 36:961–970

Macedo E, Cerqueira C, Souza D, Bispo A, Coelho R, Nascimento R (2013) Production of cellulose-degrading enzyme on sisal and other agro-industrial residues using a new Brazilian actinobacteria strain *Streptomyces* sp. SLBA-08. Braz J Chem Eng 30:729–735

Mori Y (1992) Comparison of the cellulolytic systems of *Clostridium thermocellum* YM4 and JW20. Biotechnol Lett 14:131–136

Nawel B, Said B, Estelle C, Hakim H, Duchiron F (2011) Production and partial characterization of xylanase produced by *Jonesia denitrificans* isolated in Algerian soil. Process Biochem 46:519–525

Rastogi G, Muppidi GL, Gurram RN, Adhikari A, Bischoff KM, Hughes SR, Apel WA, Bang SS, Dixon DJ, Sani RK (2009) Isolation and characterization of cellulose-degrading bacteria from the deep subsurface of the Homestake gold mine, Lead, South Dakota, USA. J Ind Microbiol Biotechnol 36:585–598

Shankar T, Isaiarasu L (2011) Cellulase production by *Bacillus pumilus* EWBCM1 under varying cultural conditions. Middle-East J Sci 8:40–45

Sheng P, Huang S, Wang Q, Wang A, Zhang H (2012) Isolation, screening, and optimization of the fermentation conditions of highly cellulolytic bacteria from the hindgut of *Holotrichia parallela* larvae (Coleoptera: Scarabaeidae). Appl Biochem Biotechnol 167:270–284

Sheng T, Zhao L, Gao L-F, Liu W-Z, Cui M-H, Guo Z-C, Ma X-D, Ho S-H, Wang A-J (2016) Lignocellulosic saccharification by a newly isolated bacterium, *Ruminiclostridium thermocellum* M3 and cellular cellulase activities for high ratio of glucose to cellobiose. Biotechnol Biofuels 9:172

Tai S-K, Lin H-PP, Kuo J, Liu J-K (2004) Isolation and characterization of a cellulolytic *Geobacillus thermoleovorans* T4 strain from sugar refinery wastewater. Extremophiles 8:345–349

Tsegaye B, Balomajumder C, Roy P (2018) Isolation and Characterization of Novel Lignolytic, Cellulolytic, and Hemicellulolytic Bacteria from Wood-Feeding Termite *Cryptotermes brevis*. Int Microbiol1–11

Wu Y-R, He J (2015) Characterization of a xylanase-producing *Cellvibrio mixtus* strain J3-8 and its genome analysis. Sci Rep 5:10521
